# Characterisation of the PS-PMMA Interfaces in Microphase Separated Block Copolymer Thin Films by Analytical (S)TEM

**DOI:** 10.3390/nano10010141

**Published:** 2020-01-13

**Authors:** Julius Bürger, Vinay S. Kunnathully, Daniel Kool, Jörg K. N. Lindner, Katharina Brassat

**Affiliations:** 1 ‘Nanostructuring, Nanoanalysis and Photonic Materials’ Group, Department of Physics, Paderborn University, 33098 Paderborn, Germanylindner@physik.upb.de (J.K.N.L.); 2 Institute of Lightweight Design with Hybrid Materials (ILH), 33098 Paderborn, Germany; 3 Center for Optoelectronics and Photonics Paderborn (CeOPP), 33098 Paderborn, Germany

**Keywords:** block copolymers, self-assembly, polymer interface, nanostructure metrology, line edge roughness LER, (S)TEM, STEM-EELS of PS and PMMA

## Abstract

Block copolymer (BCP) self-assembly is a promising tool for next generation lithography as microphase separated polymer domains in thin films can act as templates for surface nanopatterning with sub-20 nm features. The replicated patterns can, however, only be as precise as their templates. Thus, the investigation of the morphology of polymer domains is of great importance. Commonly used analytical techniques (neutron scattering, scanning force microscopy) either lack spatial information or nanoscale resolution. Using advanced analytical (scanning) transmission electron microscopy ((S)TEM), we provide real space information on polymer domain morphology and interfaces between polystyrene (PS) and polymethylmethacrylate (PMMA) in cylinder- and lamellae-forming BCPs at highest resolution. This allows us to correlate the internal structure of polymer domains with line edge roughnesses, interface widths and domain sizes. STEM is employed for high-resolution imaging, electron energy loss spectroscopy and energy filtered TEM (EFTEM) spectroscopic imaging for material identification and EFTEM thickness mapping for visualisation of material densities at defects. The volume fraction of non-phase separated polymer species can be analysed by EFTEM. These methods give new insights into the morphology of polymer domains the exact knowledge of which will allow to improve pattern quality for nanolithography.

## 1. Introduction

The self-assembly of block copolymers (BCPs) in thin films is one of the most promising approaches for next generation surface nanopatterning. A large variety of patterns with nanoscale features is accessible and can be easily created on large areas [1,2,3,4,5]. Block copolymer self-assembly is mainly driven by interfacial energies, i.e., polymer-polymer interactions of the BCP species and their interactions with a substrate or gaseous environment. During microphase separation, periodical arrays of sub-20 nm polymer domains are formed. The polymer domain shapes exhibit spherical, lamellar or cylindrical geometries determined by the polymer block length ratio [6,7,8,9,10]. These self-assembled polymer domains can then be used as templates for various purposes: removing one species selectively, one can create shadow masks for further lithographical processing [11], membranes used in nanofiltration [12,13] or versatile electrochemical devices [14]; Janus-type nanostructures can be created by microphase separation of terpolymers [15]; chemically functional polymers are exploited for domain specific diffusion for battery applications [16,17] and photovoltaics [18] or in sequential infiltration synthesis (SIS) processes [19,20]. For all these purposes the resulting pattern quality and device performance is determined by the initial morphology of the self-assembled polymer domains, i.e., the domain orientation within the film, the domain order and long range order as well as the accuracy of domain shapes.

The domain morphology is largely determined by the interface between the phase separated polymer domains. This interface between the polymer species is not sharp but interpenetration of polymer chains leads to concentration gradients. The resulting width of this interface is not negligible as it can easily exceed 40% of the actual domain size [21]. This interfacial width also strongly influences e.g., nanopattern line edge and line width roughness, which are important parameters for technological applications. The ratio between the equilibrium pattern periodicity *L_0_* and interfacial width ∆ is discussed in recent literature as being crucial to estimate the suitability of a certain BCP system for sub-10 nm L_0_ nanopatterning applications [21]. Thus, efforts are made to investigate the morphology of these interfaces and the origin of interfacial fluctuations in order to minimise interfacial widths [21,22,23,24]. New polymer species are, for instance, designed to reduce the interfacial width to <10% of L_0_ [21].

The importance of the interface morphology for understanding polymer (de)mixing is being discussed since decades. The first model aiming to describe the interface between two polymer species was introduced by Helfand [25]. He investigated the interfacial tension □ and interfacial width ∆ by mean field theory for polymers within the strong segregation limit, showing that the interface is basically determined by the Flory Huggins parameter χ and the statistical polymer segment length a:

□_∞_ = k_B_T a (χ/6)^1/2^/v (1)

∆_∞_ = 2a (6χ)^−1/2^ (2)

with v being the average monomeric volume. Similar to many other early predictions of the domain and interface morphology, this estimate assumes sufficiently large polymerisation degrees. By approximating infinite molecular weight (indicated by the subscribes ∞), effects of chain size and chain ends can be neglected [6,7,26,27]. It is to note that the interfaces between domains of block copolymers and respective blends of homopolymers are identical as was stated by the narrow-interface-approximation theory by Helfand and Wasserman [28] and experimentally verified by Shull et al. [29,30].

Later, finite-molecular weight and chain-end effects were found to decrease the interfacial tension and, thus, to increase the interfacial width [31,32]. An extended model including the chain length was proposed by Semenov in 1993 [33,34]. Since then, intensive further work on pitch scaling and interfacial width scaling in dependence of the effective Flory Huggins parameter χN (with N the degree of polymerisation) followed [35,36,37,38,39,40]. Simulations based on self-consistent field theory by C. T. Black [34] as well as A. Hannon and J. Kline [41] describe the interface width in good agreement with experimental observations

∆_x_ = ∆_∞_ {1 + [24/(χNπ^2^)]^1/3^} (3)

The broadening of the experimentally observed interface morphology compared to the simple thermodynamic model by Helfand is discussed to result from local fluctuations in the position of interfaces, thermal fluctuations of the concentration profiles, local stretching of the polymer chains [42,43] and polydispersity of polymer chains [44]. Semenov [33] describes these fluctuations as the deviation of interface positions σ in dependence of the interfacial tension, interfacial width and the polymer pattern periodicity L_0_:

σ^2^ = (2π□_∞_)^−1^ ln(L_0_∆_∞_^−1^) (4)

More recently, the interfacial width was found to be, in addition, dependent on the block copolymer annealing method [22] and the annealing temperature. The origin of these connections is the temperature dependency of the Flory Huggins parameter. It was observed that annealing near the order-disorder transition (ODT) temperature results in a larger interfacial width as the two blocks begin to mix, while the interface is sharper at annealing temperatures far below ODT. Therefore, the interfacial width is assumed to be a suitable measure for the progression of the phase separation [45].

The experimental observation and characterisation of the polymer domain morphology is demanding. Reciprocal space methods are being used to analyse order and polymer domain interfaces [45]. Measurements of X-ray and neutron reflectivity and evaluation of volume fraction profiles of the polymer species [22,40,41,42,46,47,48,49,50] or secondary ion mass spectrometry [51] are most common techniques. These methods, however, do not allow for acquisition of spatially resolved information and, for instance, neutron scattering experiments require deuteration of polymers to distinguish between the organic materials, which for many polymers is not easy in terms of synthesis and might influence the polymer behaviour. More recently resonant soft X-ray reflectivity (RSoXR) was introduced as a new method allowing for good contrast in unmodified organic materials [23,51,52,53,54]. For instance, recent work by Kline and coworkers [21] successfully applied RSoXR to determine domain periodicity and interfacial widths in high-χ block copolymers.

Real space analysis of block copolymer thin films is most commonly performed by scanning electron microscopy (SEM) and atomic force microscopy (AFM) [45]. These methods allow for the investigation of pattern order, however, resolution is limited and the interface between domains is not accessible. Transmission electron microscopy (TEM) of microphase separated BCPs is much more rarely used even though it is particularly suitable to investigate the morphology and shape of polymer domains at much higher resolution. Insightful works were published investigating polymer domain morphologies [55,56,57] and concentration profiles at polymer interfaces [44]. Recently, Segal-Peretz et al. [58,59] investigated morphologies and positional interface fluctuations of infiltrated polymer domains employing TEM tomography. Staining of one polymer domain e.g., with RuO_4_ or OsO_4_ [55,56,57] or infiltration of one polymer domain with e.g., Al_2_O_3_ [58] is most often used for contrast enhancement in these TEM studies. However, the incorporation of material for contrast improvement holds several disadvantages. It always leaves doubt on artefacts introduced with the foreign material due to chemical (cross-linking or chain scission reactions) or physical modifications (contraction or expansion of domains) [44]. It is also not fully understood how and where materials are infiltrated at domain interfaces where polymer chains can interpenetrate or form a concentration gradient [58]: Infiltration might reconstruct a material domain up to a certain threshold concentration or within all material volume containing any fraction of the distinct material. This will largely influence the apparent domain size and shape. In addition, selective staining is not easily available for all kinds of polymers.

To our knowledge, no high-resolution real space imaging of unmodified self-assembled polymer domains in block copolymer thin films has been published so far. This is probably due to many factors, including the difficulty to obtain reasonable contrast between the polymer species at electron energies above 100 keV where in the past TEMs used to have sufficient resolution, and the sensitivity of polymers to irradiation with energetic electrons. Attempts have been made to exploit phase contrasts induced between polymers by using a strong objective lens defocus, however, on the expense of spatial resolution [60]. Current analytical electron microscopes, equipped with correctors for spherical lens aberrations, and fast detectors can overcome these limits. Thus, in this work, we investigate the domain morphology and interface of unmodified unstained microphase separated polystyrene-*b*-polymethylmethacrylate (PS-*b*-PMMA) thin films by analytical (S)TEM. We investigate PS-*b*-PMMA BCPs with different block length ratios forming PMMA cylinders in PS (PS:PMMA 70:30), PS cylinders in PMMA (PS:PMMA 30:70) or alternating PS and PMMA lamellae (PS:PMMA 50:50).

We characterise the interface between PS and PMMA nanodomains in the block copolymers and correlate data from theory and literature on domain size, interface position and interfacial width to high resolution real space images. In particular, we image the polymer domain morphology and their interface using STEM revealing the internal structure of polymer domains, the positional fluctuation of the interfaces as well as the occurrence of grains enlarging the line edge roughness—all features which are not accessible by other techniques. STEM electron energy loss spectroscopy is used to investigate the chemical composition of polymer domains exploiting plasmonic resonances of the polymers as well as the near edge fine structure at their carbon K-edge. High-resolution imaging at the plasmon resonance and zero loss imaging are applied using energy filtered TEM spectroscopic imaging. Finally, energy filtered TEM thickness maps are shown to visualise the periodical density variations of the polymer domains revealing e.g., the polymer composition at defects in the polymer pattern.

## 2. Materials and Methods

### 2.1. Materials and Sample Preparation

Three different polystyrene-*b*-polymethylmethacrylate (PS-*b*-PMMA) block copolymers with block length ratios of PS:PMMA of 70:30, 30:70 and 50:50 were purchased from Polymer Source Inc. and dissolved in toluene (analytical grade, *C. Roth GmbH*). The molecular weights of the different polymers can be found in Table 1. The polydispersity indices are between 1.06–1.09. Thin films of the different block copolymers were spin casted onto silicon wafers covered with a thermally grown 700 nm thick silicon oxide sacrificial layer. For all BCPs, the oxide was functionalized with 5–7 nm thick PS-*co*-PMMA random copolymer brushes (M_n_ = 5.2–8.5 kg/mol, 58–66 mol% PS content) from Polymer Source Inc. BCP films with thicknesses of 35 nm for both, the PS:PMMA 70:30 and the PS:PMMA 30:70 BCP, and of 40 nm for the PS:PMMA 50:50 BCP were thermally annealed at 180 °C at a pressure of 10^−7^ mbar for 24 h to enable microphase separation. The long annealing time was chosen to ensure complete microphase separation. It was shown that the initial phase separation is an extremely rapid process which is only followed by a slow pattern optimisation through defect annihilation [45]. The long annealing of 24 h, thus, should allow for a terminated phase separation.

To obtain free-standing BCP membranes for TEM analysis, sample preparation was performed as described previously [11]. Briefly, microphase separated BCP films were released from the substrate by etching of the thick sacrificial silicon oxide layer in 10% HF_aq_ at room temperature. The floating BCP membrane was then skimmed off the etchant with a TEM grid. In this work, Quantifoil on Au grids purchased from Plano GmbH were used. Comparison of AFM images taken from the BCP film prior to and after HF dipping (not shown here) confirmed that the diluted HF solution of moderate temperature does not affect the polymers.

### 2.2. Characterisation Techniques

Scanning electron microscopy (SEM) images were taken with a Zeiss ultra plus at an acceleration voltage of 2 kV with an in-lens detector. Atomic force microscopy (AFM) was performed using a Digital Instruments Dimension 3100 in non-contact mode with 65 kHz Al-coated cantilevers (MikroMasch) with nominally 8 nm tip diameter.

Analytical (scanning) transmission electron microscopy ((S)TEM) was performed using a JEOL JEM-ARM200F equipped with a cold field emission electron gun (CFEG) and a probe-side mounted ASCOR C_s_-corrector (CEOS GmbH) allowing for the correction of geometric aberrations up to the 5th order and therefore resolution in the STEM mode of better than 1 Å at an acceleration voltage as low as 60 kV. All images shown in this work are acquired at this voltage where no beam damage, i.e., chemical or structural changes due to high-energy radiation, was observed. For comparison, images obtained at 200 kV can be found in the Supplementary Information Figure S1. All TEM images are acquired with 150 µm condenser lens aperture and captured on a 4 k × 4 k GATAN OneView camera. STEM images are recorded with a convergence semi-angle of 16.6 mrad on an annular dark field (ADF) detector, which collects scattered electrons at polar collection semi-angles of 69–147 mrad, using a 40 µm condenser lens aperture and a camera length of 12 cm.

Analytical methods such as energy filtered TEM (EFTEM) spectroscopic imaging (EFTEM-SI) and electron energy loss spectroscopy (EELS) are applied for differentiation between PS and PMMA domains. EFTEM and EELS are conducted with a GATAN GIF-Quantum ER image filter and are captured on a 2 k × 2 k CCD camera (GATAN UltraScan). Operating the CFEG at maximum beam current, the energy resolution as expressed by the zero-loss peak FWHM was 0.65–1.1 eV at 60 keV. Overview EEL spectra are generated with a broad beam in TEM mode, for spatially resolved analysis STEM-EELS line-scans were performed. EELS spectra are recorded in dual EELS mode with a dispersion of 0.1 eV for low-loss (−20 eV to 184.8 eV) and high-loss (200 eV to 404.8 eV) parts of the spectra. EFTEM thickness maps are obtained using the t/λ-method and mean free paths λ, calculated according to Iakoubovskii et al. [61].

### 2.3. Calculation of Expected Interfacial Widths and Interface Position Fluctuation

The theoretically expected interfacial widths for the polymers used in this work were determined using the models by Helfand and Kline, respectively. The Kuhn lengths, i.e., the statistical polymer segment lengths, of PS and PMMA are both approximately a = 0.70 nm [34].

As stated above, the Flory Huggins parameter is temperature dependent following

χ(T) = χ_s_ + χ_H_/T (5)

with χ_s_ and χ_H_ being the entropic and enthalpic terms of the Flory Huggins parameter. For PS-PMMA these contributions were determined [62] to

χ_PS-PMMA_ = 0.028 + 3.9/T (6)

In this work, the Flory Huggins parameter for a temperature of 180 °C is used as this is the annealing temperature enabling microphase separation in the presented experiments.

The minimum interfacial width for a PS-PMMA interface according to the model by Helfand (Equation (2)) can be determined to

∆_∞_ = 2.99 nm

The interfacial widths according to the model by Kline et al. (Equation (3)) for the three PS-*b*-PMMA block copolymers used in this work are collected in Table 1. This model takes the polymerisation degree of the polymer blocks into account. Thus, the resulting interfacial width is larger than predicted by Helfand. However, as the polymerisation degrees and molecular weights of the three polymers are comparable, the expected interfacial widths only exhibit small differences. This is also in accordance to experimental investigations applying neutron scattering by Anastadiadis [46] who found similar interface widths at interfaces of polymers with molecular weights between M_n_ = 30–300 kg/mol.

The deviation of the interface position σ was determined according to Equation (4) with the interfacial tension □_∞_ according to Equation (1) and the interfacial width ∆_x_ after the model by Kline et al. (Equation (3). For simplification, the monomeric volume *v* was related to the statistical segment length *a* assuming that the segments occupy a spherical volume:

v = 0.52 a^3^

The positional fluctuation σ is usually directly correlated to the line edge roughness (LER). The results are assembled in Table 1.

## 3. Results

**Figure 1:**
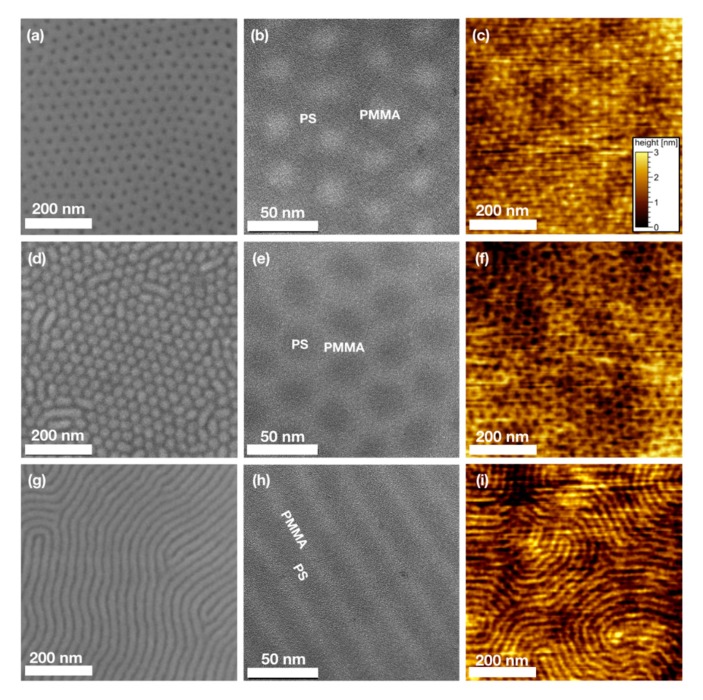
SEM, AFM and TEM images of the three polystyrene (PS)-b-PMMA block copolymers (BCPs) with block length ratios of (**a**–**c**) PS:PMMA 70:30, (**d**–**f**) PS:PMMA 30:70 and (**g**–**i**) PS:PMMA 50:50, respectively. (**a**,**d**,**g**) show SEM images, (**b**,**e**,**h**) display bright field TEM images and (**c**,**f**,**i**) are AFM height images with same height scales as in (**c**). In all images, both polymer species are apparent and no staining or other sample treatment was used.

Figure 1 gives an overview of the three PS-*b*-PMMA block copolymers investigated in this work. Each row shows a different polymer with a block length ratio of PS:PMMA 70:30 (a–c), PS:PMMA 30:70 (d–f) or PS:PMMA 50:50 (g–i), respectively, investigated by scanning electron microscopy (SEM, left column), bright-field transmission electron microscopy (TEM, middle column) and atomic force microscopy (AFM) (right column). In all images both microphase separated PS and PMMA domains are shown after annealing, no polymer species was selectively removed or modified. SEM and AFM images were recorded with the BCP films supported by the substrate, while for TEM free standing membranes were used.

SEM and AFM are the most commonly used techniques to analyse block copolymers. Domain size d and pattern periodicity L_0_ are commonly determined from SEM images using grey scale thresholding techniques and image analysis based on Delaunay triangulation [5,63]. For the polymers investigated here, domain sizes and periodicities derived from SEM images are collected in Table 1. AFM imaging allows to determine height differences between the polymer domains. Figure 1c,f,i shows elevated PMMA domains with a difference of 1.1–1.3 nm to the PS domains.

TEM bright-field images of the three BCPs are shown for comparison. A contrast between PS and PMMA domains is visible in these unstained polymers, even though chemical compositions and densities of PS and PMMA are similar. While the resolution of SEM hardly allows to judge on the shape and lateral extension of polymer interfaces and AFM imaging is limited by the cantilever dimension, high-resolution TEM allows to investigate the polymer domain morphology close to the atomic scale. Thus, the exact shape of polymer domains as well as the blurred interface between distinct polymer domains become visible only here. This advantage will be employed in detail in the following using analytical TEM as well as STEM.

### 3.1. Polymer Domain Morphology and Line Edge Roughness Investigated by STEM-ADF

**Figure 2:**
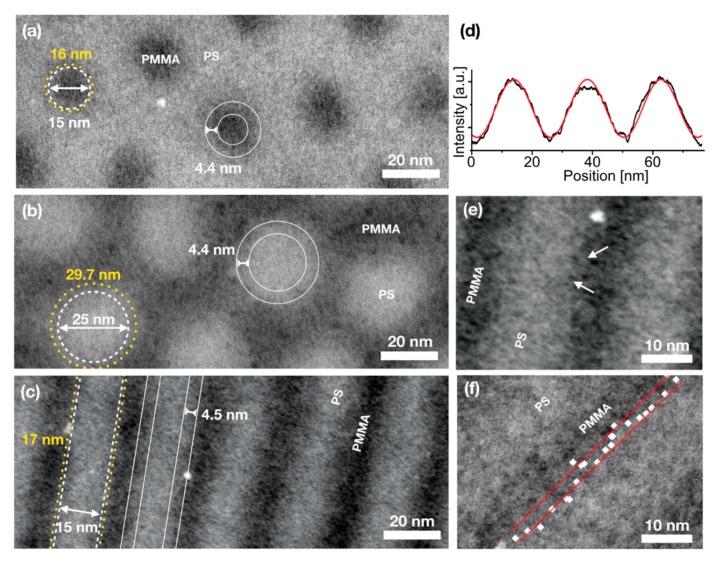
STEM-annular dark field (ADF) images of PS-*b*-PMMA BCPs with block length ratios of (**a**) PS:PMMA 70:30, (**b**) PS:PMMA 30:70 and (**c**) PS:PMMA 50:50 with bright contrast of PS and darker contrast of PMMA. Sketches in these images mark the domain sizes as measured by SEM (white dashed lines), domain sizes determined at 50% contrast in STEM ADF (yellow dotted lines) and theoretical interfacial widths of 4.4 and 4.5 nm (white solid lines). (**d**) Contrast profile perpendicular to alternating PS and PMMA lamellae as in (**c**) (black) superimposed by a sinusoidal fit (red). (**e**) Image section of BCP PS:PMMA 50:50 at higher magnification. White arrows mark grains of opposite contrast which might contain entrapped foreign polymer. (**f**) Image section of BCP PS:PMMA 50:50 with identified positions of 50% contrast (white dots) and resulting line edge roughness (LER) (distance of red lines = 3.2 nm).

Figure 2 presents scanning transmission electron microscopy (STEM) images of the three unstained PS-*b*-PMMA block copolymers acquired with an annular dark field (ADF) detector. Dark areas correspond to PMMA domains while PS gives a bright contrast. Figure 2a shows PMMA cylinders in a PS matrix formed by BCP PS:PMMA 70:30, Figure 2b shows the inverse pattern, i.e., PS cylinders in a PMMA matrix (BCP PS:PMMA 30:70) and Figure 2c displays alternating PS and PMMA lamellae formed by the BCP PS:PMMA 50:50. It is to note that these contrasts, PS appearing brighter than PMMA, are not as expected. Heavier atoms should appear bright in dark-field imaging, thus, PMMA would be expected to give a brighter contrast compared to PS. The observed material assignment was, however, also reported by others [58] and is further verified by EFTEM thickness and elemental mapping (Section 3.4 and Section 3.5).

The STEM-ADF images clearly show that the interface between PS and PMMA domains is not sharp but exhibits a broad, blurred material contrast between polymer species. The absence of sharp interfaces between polymer species in the STEM images is mainly due to the absence of sharp interfaces in the BCP film. The crucial influence of the imaging technique on the interpretation and analysis of the polymer domain morphology becomes apparent when comparing feature sizes determined from SEM and STEM-ADF images. In order to highlight this difference sketches of the domain sizes as measured from SEM images of these exact polymers are added in Figure 2a–c as white dashed lines (the positions of these sketches are estimated, the rings were centred around the middle of cylinders). These domain boundaries seem to cut the blurred broad interfaces at arbitrary positions, as no sharp interface is visible.

Thus, the identification of the interface position and domain size from STEM images is not obvious. One reasonable way to determine an average interface position is shown exemplarily for the lamellae-forming BCP. Figure 2d shows a line plot of the contrast cutting perpendicularly through three parallel lamellae of alternating PS and PMMA as in Figure 2c. The contrast distribution and thus the composition profile follows a sinusoidal curve (red fit). This is in good agreement with resonant soft X-ray reflectivity (RSoXR) observations by Sunday et al. [21] who found this sinusoidal material distribution being specific for BCPs with a comparably low χ and χN ≲ 23, as apparent in PS-*b*-PMMA BCPs (Table 1). Figure 2d shows, that no plateau is formed in the composition profile, thus, no regime of completely separated pure phases is apparent. Thus, an experimental determination of the width of the domain interface is not feasible. Theoretical values of the interfacial width were determined following Equation (4) (Table 1) to 4.4–4.5 nm and are marked in the STEM-ADF images in Figure 2 for illustration (white solid lines). In literature, the width of PS-PMMA interfaces in similar BCPs is determined by neutron scattering to 5 nm [22]. If one considers the position of the domain interface at 50% concentration of one or the other polymer species, i.e., a locally predominant composition of either PS or PMMA, it is possible to estimate the position of the interface. The resulting average domain sizes designating the 50% concentration threshold are listed in Table 1 and marked in Figure 2a–c by yellow dotted lines. The resulting domain sizes differ from those determined by SEM showing that SEM underestimates the PS domain sizes.

The high-magnification STEM images show an additional internal structure of the polymers indicating strong compositional fluctuations along the lamellae as well as within or around cylinders. This internal structure is not accessible with SEM, AFM or other techniques. These fluctuations most likely result from the interfacial width and positional fluctuations of the domain interface along the long-axis of the lamella as well as along their through-film dimension. It was shown by Segal-Peretz et al. [58,59] using TEM tomography on stained PS-*b*-PMMA cylinders that the domain morphology through the polymer film is strongly distorted compared to a perfect cylinder. In all (S)TEM images shown in this work one analyses the projection of all these positional fluctuations.

One more striking observation is the existence of sharply bordered grains of opposite contrast within domains close to their 50%-interface. Such grains are shown in Figure 2e in higher magnification and some are exemplarily marked by arrows. As contrasts are assigned to different materials and as these grains appear in both polymer domains, it is likely that they contain the opposite material trapped within the foreign domain. Such nanoscale domains could form when polymer chains are trapped with contrariwise chain orientation not able to overcome the energy barrier to reorient during microphase separation, or with polymer chains being fully incorporated with both ends inside one polymer domain. In order to minimise interfacial areas, the entrapped parts of chains will then form polymer coils. This hypothesis is further supported by the observation that the frequency is much lower at the polymer domain interfaces of cylinder-forming BCPs than at those of the lamellae-forming BCP. From a geometrical point of view, the release of polymer ends within the wrong polymer domain, i.e., the healing of such defects, is favoured at curved interfaces compared to planar interfaces as the likelihood for the chains to reach an interface in close proximity is larger at curved than planar interfaces. It is also possible that the grains are agglomerates of random copolymer chains which were released from the functionalized SiO_2_ substrate during polymer membrane preparation by HF etching. Such patches could adhere to the BCP film or be redeposited during the skimming of the polymer membranes with the TEM grid. However, this interpretation cannot convincingly explain the predominantly inverted contrast of these grains compared to their surroundings.

All these observed features at the polymer domain interfaces (interface width, positional fluctuations, grains) are expected to contribute to in the line edge roughness (LER) of domains. The LER was determined analysing the positional fluctuations of the 50% concentration threshold at several positions along a domain boundary. If one identifies the midpoint between absolute minima and maxima in several line scans, as in Figure 2d, along one domain one can determine the positional interface fluctuation, i.e., the LER. In case of the lamellae (PS:PMMA 50:50), the LER amounts to 1.66 ± 0.46 nm. This value is of the same order of magnitude as the theoretically expected value of 0.93 nm (Table 1) determined using Equation (5). The larger value can be explained by the superposition of interface fluctuations not only along one lamella but also along the domain interfaces through the polymer film. As stated above, it was observed in Reference [59] that the polymer domains are not perfectly cylindrically shaped but their morphology is irregular. It was also shown that the fluctuations of the interface position become even stronger towards the substrate and towards the interface with air/vacuum than they are within the polymer film. As our investigations are based on the projection of the domain interfaces throughout the film, a larger LER is expected. However, an LER of 1.66 nm can typically not be found on nanopatterns created using similar BCP lamellae as lithography template. LER of replicated patterns are usually much larger and measure approximately 4.8 nm [64]. It is likely that the larger LER results from the grains found close to the domain interfaces (Figure 2e). One can include these grains into the determination of the LER by not measuring the midpoint between absolute minima and maxima in the line plot (which neglects the existence of these grains), but defining the transition from >50% to <50% intensity as interface position. These positions are marked by white dots in Figure 2f. If one includes these grains in this way, the positional fluctuation doubles and measures 3.2 ± 1.9 nm. This observation also supports that the grains are no artefacts from sample preparation but features within the microphase separated polymer film.

In case of cylinder-forming BCPs (PS:PMMA 70:30 and 30:70), the large grains occur to a smaller extend, i.e., they poorly influence the line edge roughness of domains. The LER of these cylinder-forming BCPs can be determined to 1.78 nm for the BCP PS:PMMA 70:30 in Figure 2a and to 1.29 nm for the BCP PS:PMMA 30:70 in Figure 2b. Again, it is assumed that the curvature of the domains allows for the formation of smoother domain interfaces.

### 3.2. Spatially Resolved Investigation of the Composition of the Polymer Film Using (S)TEM-EELS

Electron energy loss spectra (EELS) were measured in TEM mode as well as STEM mode to investigate the chemical composition of assigned PS and PMMA domains. Spectra were collected using dual-EELS detection allowing for high integration times as well as precise determination of energies.

**Figure 3:**
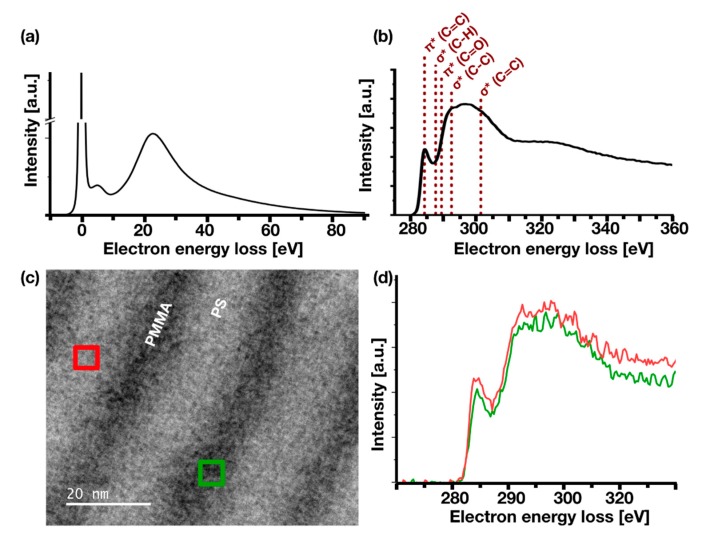
TEM-electron energy loss (EEL) spectra of BCP PS:PMMA 50:50 showing (**a**) the low-loss region and (**b**) high-loss at the carbon K-edge with assignment of peaks in (**b**) according to References [65,66,67,68]. (**c**) STEM-ADF image with marked positions of corresponding STEM-EELS spectra in (**d**). The spectra are displayed after zero-loss peak alignment and background subtraction.

Combined EEL spectra of PS and PMMA taken from the 50:50 PS:PMMA BCP in the low-loss region and the high-loss region are shown in Figure 3a,b, respectively. These spectra were taken in TEM mode at 60 kV, the zero loss peak having a FWHM of 0.65 eV. In the low-loss region (Figure 3a), two distinct peaks are detected at 7 eV and at approximately 21 eV energy loss. The peak at 7 eV can be assigned to a π–π* excitation of electrons in the aromatic ring of PS [65,66] verifying the existence of intact PS. The bulk plasmon peaks of PS and PMMA are found at 22 eV and 20 eV, respectively [67,68], appearing as a broad peak around 21 eV loss in this spectrum. Figure 3b shows the high-loss region of the EEL spectrum at the carbon K-edge. The near-edge fine structure of the C-K edge allows to differentiate between carbon binding states. In particular, the distinct peak at 285 eV loss corresponding to a C1s→π* (C=C) transition [65] again provides evidence of the presence of polystyrene, since a C=C bond does not exist in PMMA and thus PMMA does not show such a peak [67], while C-H bonds and C-C bonds can be found in both, PS and PMMA.

STEM-EELS was employed to investigate the local distribution of PS and PMMA in the phase separated system. Figure 3c shows a STEM-ADF image with marked positions for the acquisition of the STEM-EEL spectra in Figure 3d. Spectra are very similar, which supports the hypothesis that no pure polymer domains are formed as already indicated by the sinusoidal contrast distribution found in STEM-ADF images (Figure 2d). The spectra in Figure 3d show clearly that PS exists at each sample position since the specific C1s→π* (C=C) transition at 285 eV can be found in both spectra of a PS domain and a PMMA domain. This verifies that no pure PMMA domains are present.

### 3.3. EFTEM Spectroscopic Imaging of PS and PMMA Lamellae

**Figure 4:**
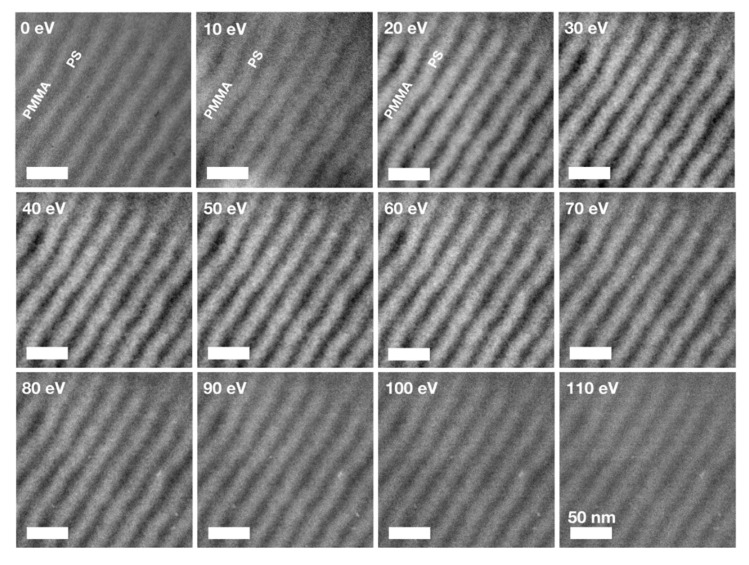
Energy-filtered TEM spectroscopic imaging of lamellae-forming BCP PS:PMMA 50:50. Images are collected from energy windows between −5 eV and +115 eV with an energy filter slit width of 10 eV. Energies noted in each image refer to the window centre. All scale bars are 50 nm.

EFTEM spectroscopic imaging was performed to identify energy windows particularly suitable for the investigation of the PS and PMMA domains. Figure 4 displays a tableau of images collected using energy windows between −5 eV and +115 eV. Each image was collected using a filter slit width of 10 eV; the energy centre is noted in each image. While at higher electron energies typically smaller slit widths are used in EFTEM-SI, at 60 keV the slit width of 10 eV allowed for images without visible distortions. Two energy windows were found to be particularly suitable for high-resolution and high-contrast imaging of the PS and PMMA domains:

- **0 eV.** EFTEM zero loss imaging is known to increase contrasts in copolymers by removing inelastically scattered electrons from the image. This technique was introduced by Kunz et al. [69] and applied to copolymer blends [44]. Compared to conventional bright-field TEM images as shown in Figure 1h, the contrast in this image is increased and the resolution is high allowing for imaging of the internal structure of the polymer domains. This is also clearly visible in the zero loss images of both cylinder-forming BCPs (Figures S2 and S3). It is to note that an inversion of the contrasts between PS and PMMA in this zero-loss region occurs: PMMA appears brighter in TEM bright-field and zero-loss filtered EFTEM images than PS. In energy filtered images with the energy window centred at values between 10 and 110 eV PS rich domains appear brighter than their PMMA rich surroundings. This contrast inversion most likely results from a comparatively large plasmon excitation in PS compared to PMMA, since the low-loss region between 10 and 110 eV is dominated by the plasmon peak as visible in Figure 3a.
- **20–60 eV.** This energy region around and above the plasmon resonances of PS and PMMA [66,67,68] gives the best material contrasts. The internal structure of polymer domains with grains of inverse contrast, as discussed on the STEM-ADF images in Figure 2, become particularly visible in images obtained at 25–44 eV. The presence of ‘bright’ grains in a darker PS surrounding can be interpreted as the presence of PMMA inclusions leading to a locally enhanced plasmonic energy loss. Vice versa, the presence of ‘darker’ grains in an environment of bright PMMA rich surrounding would indicate a lack of PMMA material due to the inclusion of small PS grains. However, it is not possible to exclude that such ‘inclusions’ are actually located at the surface and are residuals of the random copolymer brush layer. In any case, it is likely that these grains contain the opposite polymer species.

Between the two regimes highlighted above a point of contrast inversion is found around 10 eV. Here, the contrast between polymer domains is poor and the PS domains appear very broad. At higher energies from 65 eV up to 115 eV contrasts are vanishing.

A video showing a series of images through the whole energy spectrum allows for direct comparison of the contrasts and can be found in the Supplementary Information. Tableaus of images of the two cylinder-forming block copolymers PS:PMMA 70:30 and PS:PMMA 30:70 obtained at these energy windows can be found in the Supplementary Information Figures S2 and S3, respectively.

### 3.4. Determination of Material Density Distributions by EFTEM Thickness Mapping

**Figure 5:**
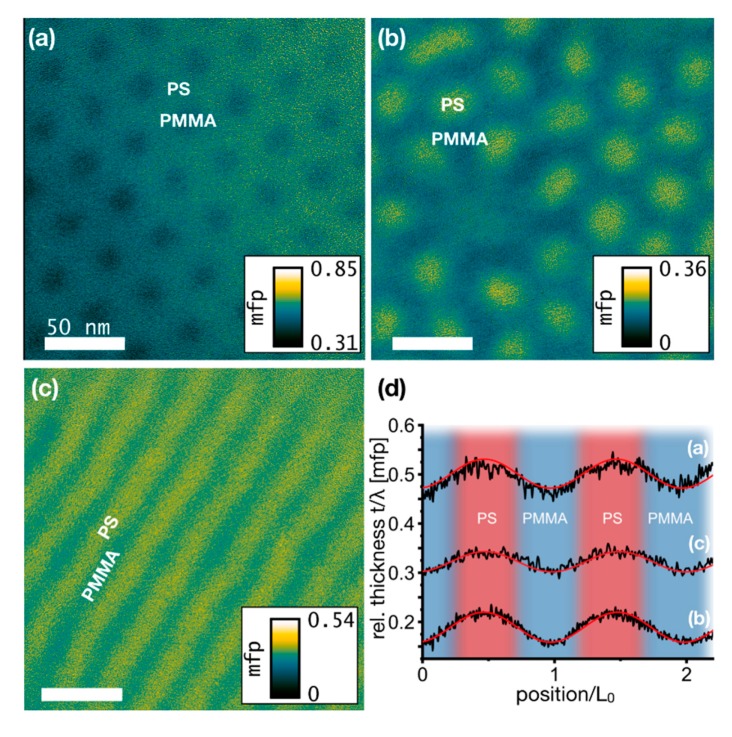
Energy-filtered TEM thickness maps of three BCPs with block length ratios of (**a**) PS:PMMA 70:30, (**b**) PS:PMMA 30:70 and (**c**) PS:PMMA 50:50. Colour coded maps display film thickness in units of mean free path (mfp). Lateral scale bars are all 50 nm. The small gradient in (**a**) from the lower left to the upper right corner is due to bending of the free polymer membrane close to a hole in the supporting Quantifoil film. (**d**) Line plots across polymer domains (position normalised to L_0_ of each polymer).

Thickness maps determined by energy-filtered TEM (EFTEM) are shown in Figure 5 for the BCPs (a) PS:PMMA 70:30, (b) PS:PMMA 30:70 and (c) PS:PMMA 50:50. Images were acquired at 60 kV with an integration time of 1.2 s/frame. Maps of the thickness *t* were measured using the t/λ-method [70], i.e., comparison of intensities in an unfiltered image and a filtered image with a 10 eV slit width at zero energy loss allowing only elastically scattered electrons to be detected. Consequently, the maps show the thickness in units of projected mean free paths λ (mfp) of electrons through the polymer domains. Using the model by Iakoubovskii [61], one can estimate the mfp at a given material mass density. The densities of PS and PMMA are assumed to 1.052 g/cm^3^ and 1.159 g/cm^3^, respectively [71]. The mean free paths then translate to 85.6 nm in PMMA and 87.6 nm in PS, i.e., they differ by 2.3%.

In all three polymers, the PS domains exhibit a larger thickness than the PMMA domains (Figure 5). Thus, for all three polymers thickness profiles across polymer domains show a periodical behaviour (Figure 5d). For better comparison, the position axis is given in units of L_0_ and curves are shifted such that the maxima match their positions. The local variation of t/λ along polymer domains follows a sinusoidal trend as was determined from contrast evaluation in STEM-ADF images in Figure 2d. If one takes into account that the mean free path of electrons is slightly larger in PS than in PMMA it becomes obvious that the thickness oscillations of block copolymer membranes are even more pronounced than those of t/λ. The average film thicknesses differ for the three polymers and amount to 43, 17 and 28 nm for the 70:30, 30:70 and 50:50 PS:PMMA films, compared to the targeted thicknesses of 35, 35 and 40 nm, respectively. Height differences between PS and PMMA domains are similar in both cylinder-forming BCPs, measuring 5.2 ± 0.2 nm. In case of the lamellae-forming BCP the height difference is with 3.5 nm significantly smaller, which might result from its smaller χN compared to the cylinder-forming BCPs (see Table 1) leading to a less efficient phase separation, thus, a stronger interpenetration of polymer chains into the opposite domain, as well as a larger density of entrapped grains of opposite polymer species (as shown in Figure 2e).

The local height differences between PS and PMMA domains appear inverted to those measured by AFM, where PMMA exceeds the PS level. It was, however, shown by Pérez-Murano and coworkers [72] that the larger elastic modulus of PMMA compared to PS can lead to such measurement artefacts during tapping mode AFM analysis of thin polymer films. It is also to note that the mass densities ρ used for the calculation of absolute polymer thicknesses are determined from bulk polymers. A different material density in microphase separated domains compared to bulk material could be apparent due to geometrical considerations of polymer chain configuration. According to the Iakoubovskii model, the mean free path *λ* depends on the mass density ρ as λ ~ ρ^−0.3^, and therefore mass densities ρ influence the conversion of measured maps to thickness maps.

Thus, assuming that the polymer film thickness is known, the EFTEM t/λ mapping method allows to visualise the density distribution within the phase separated polymer film and therefore gives further insight into the polymer mixing behaviour.

The PS matrix of the PS:PMMA 70:30 in Figure 5a, for instance, reveals a homogeneous t/λ distribution without any local variations. Due to geometrical considerations one could expect PS chains being less dense at triple points between three PMMA cylinders compared to the area between two neighbouring PMMA cylinders. This is, however, not found here, i.e., polymer chains most likely stretch, bend and compress to form a homogeneous PS matrix with minimal density fluctuations of less than 3.5%.

The image section of a PS:PMMA 30:70 BCP in Figure 5b shows a defect in the PS cylinder assembly: One PS cylinder is missing in the lower left part. This missing cylinder has a 7-fold coordinated environment to its nearest neighbours. The map reveals a slightly larger relative thickness t/λ at the expected position of the missing cylinder than in the surrounding PMMA matrix. This refers to a larger material density and/or thicker film at this exact position than in the PMMA matrix, indicating a larger PS concentration than in the surrounding PMMA matrix. Additionally, regions of smaller t/λ connect this spot to the neighbouring PS cylinders. Again, these connection lines are likely to contain increased PS concentrations. This polymer distribution suggests an insufficient microphase separation and a defect which is trapped in a metastable configuration within the process of forming distinct polymer domains.

### 3.5. Fraction of Non-Separated Polymers

**Figure 6:**
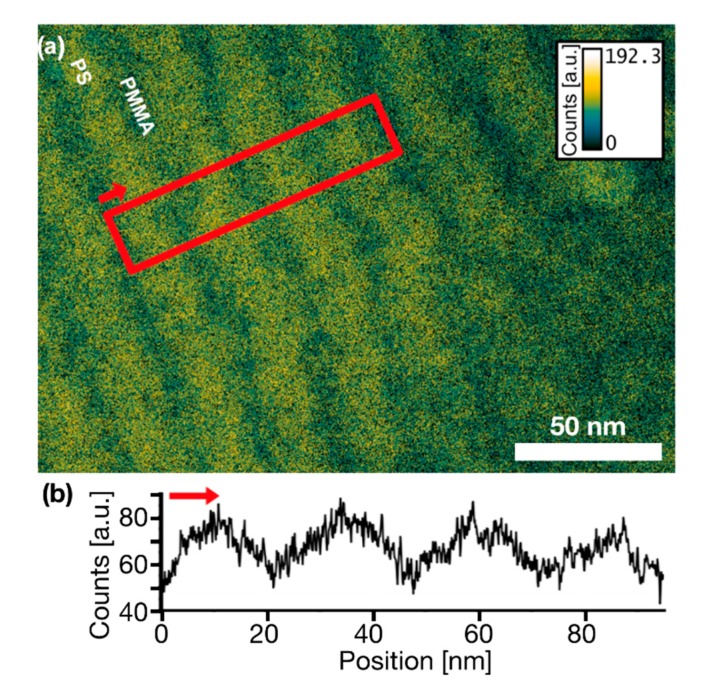
(**a**) Energy filtered TEM (EFTEM) carbon map and (**b**) concentration line-plot corresponding to the red marked area. The arrows display the direction of the line-plot.

The projected amount of any elemental species (including carbon) present at each position in a sample can be quantitatively determined using the EFTEM three-windows technique [73]. In this technique, an elemental map is calculated from three images, of which two are taken at different energies below a characteristic energy edge (for background calculation) and one in an energy window above the characteristic energy edge. After subtracting the extrapolated background from the post-edge image one obtains an image where the intensity is proportional to the number of atoms present in the sample integrated over the specimen thickness. Figure 6a displays such an EFTEM carbon map of the lamellae-forming BCP PS:PMMA 50:50 using the characteristic carbon K edge. Figure 6b shows a linear carbon concentration profile measured perpendicularly to the polymer lamellae as indicated by the red box. Since the ratio of molar carbon concentrations in pure PS and PMMA is 2:1, one would assume the concentration profile to exhibit oscillations with amplitudes of the same ratio, or more precisely with a ratio of 2.27:1, if one takes the different thicknesses of PS and PMMA lamellae (see Section 3.4) into account. Obviously, the projected carbon concentration oscillates by a much smaller amount, which can be attributed to the intermixing between PS and PMMA. Assuming that the volume fractions φ of PS inclusions in PMMA domains and of PMMA inclusions in PS domains are identical for the 50:50 BCP, one can calculate a volume fraction of φ = 20% of inclusions of the opposite polymer in the centre of each polymer lamella. Details of the calculation are given in the Supplementary Information.

## 4. Discussion

Self-assembled block copolymer nanostructures, which are widely used as templates for next generation nanolithography, were analysed by analytical (scanning) transmission electron microscopy ((S)TEM). In this work, cylindrical and lamellar nanodomains of PS and PMMA in microphase separated BCP thin films are imaged without staining at highest resolution using electrons at an energy as low as 60 keV. In contrast to more commonly used reciprocal space methods, real space imaging using analytical (S)TEM allows to correlate the internal structure of the polymer domains with characteristic parameters of these polymer patterns such as domain size, interface width and line edge roughness.

In particular, STEM dark-field images are presented, which reveal the internal structure of PS and PMMA domains. A sinusoidal contrast distribution along polymer domains allows to conclude that no pure domains containing one single polymer species are present but that periodical concentration gradients form the assigned PS or PMMA domains. This poor polymer separation might be due to the small Flory-Huggins parameter of PS and PMMA as well as the presence of a thick random copolymer brush locally promoting [74] and appearing like polymer intermixing. Thus, the term of an ‘interfacial width’ must be used carefully for the popular PS-*b*-PMMA BCPs. Line edge roughnesses (LER) were determined estimating the positional fluctuations of an interface at 50% polymer composition. One might assume that the LER measured in the projections of STEM or TEM images are affected by a possible tilting or wiggling of domain boundaries through the film. However, the isotropic smearing of interfaces around cylindrical domains in all directions suggests that such wiggling occurs only on a molecular level. Domain wiggling is observable in lamellar BCP films in in-plane directions, but on a wavelength which is large compared to the BCP film thickness. Therefore, such wiggling should not add very much to the LER observed. It is shown, however, that polymer grains, found particularly frequently in the lamellae-forming polymer, contribute largely to the line edge roughness of nanodomains. Electron energy loss spectroscopy (EELS) and energy filtered TEM (EFTEM) spectroscopic imaging were applied to investigate the chemical composition of the polymer domains. Acquiring images of polymer domains using electrons with a particular energy loss close to the bulk plasmon peak, allows for high-resolution imaging of the unstained polymers with strong material contrasts. EFTEM thickness maps were acquired revealing the density and film thickness distribution of phase separated BCPs giving insights into the spatial polymer distribution e.g., at defects in the polymer pattern. For the lamellae-forming BCP it is shown that the degree of microphase separation can be determined by analytical (S)TEM. It is found that even for the long-term annealing conditions applied here, a minimum of 20% volume fraction of non-separated polymer species is contained in the microphase separated lamellae, while in the case of cylinder-forming BCPs the fraction might be lower due to geometrical advantages.

Our analytical (S)TEM investigations shed light on the internal structure of polymer domains and polymer domain morphology, which impact pattern replication for lithography and infiltration purposes directly. These insights help elucidate the origin of line edge roughnesses of replicated nanopatterns and the limits in accuracy of selective infiltration as well as transport mechanisms in functional BCPs. It will be interesting to apply analytical STEM to investigate the properties of homopolymer blends or BCP-homopolymer blends which are often used to improve pattern order. Promising findings can also be expected when investigating other BCP species than PS-*b*-PMMA, such as high-*χ* polymers used to form sub-10 nm pitch patterns, where polymer segregation is more efficient and thus pure polymer domains with a smaller interfacial width are expected. Such high-*χ* polymer often include one Si-containing polymer species, thus, it can be anticipated that imaging with the above shown techniques will be even more facile.

## Figures and Tables

**Table 1:** Overview of polymer specifics. Molecular weights M_n_ (PS-PMMA), polymerisation degrees N_PS_-N_PMMA_, product of Flory Huggins parameter and polymerisation degree χN, domain size d_SEM_ and periodicity L_0__,SEM_ determined from SEM images, domain size d_TEM_ determined by TEM, height differences between polymer domains h_AFM_ determined by atomic force microscopy (AFM) (with polymethylmethacrylate (PMMA) domains exhibiting a larger thickness), interfacial widths ∆_x_ after Hannon/Kline and positional fluctuation of interface σ.

Polymer	M_n_ (PS-PMMA) [kg/mol]	N_PS_-N_PMMA_	χN	d_SEM_ [nm]	L_0,SEM_ [nm]	d_TEM_ [nm]	h_AFM_ [nm]	∆_x_ [nm]	σ [nm]
PS:PMMA 70:30	46.1–21.0	443–210	23.9	15.0± 2.6 ^a^	35.0 ± 4.4	16.0 ± 0.9 ^a^	1.12 ± 0.30	4.39	1.04
PS:PMMA 30:70	20.2–50.5	194–505	25.6	24.6± 4.5 ^b^	35.6 ± 6.0	29.7 ± 0.6 ^b^	1.15 ± 0.27	4.36	1.04
PS:PMMA 50:50	25.0–26.0	240–257	18.2	14.7 ± 2.4 ^c^	24.3 ± 1.2	9.2 ^c^	1.28 ± 0.28	4.53	0.93

^a^ Diameter of PMMA cylinder; ^b^ diameter of PS cylinder; ^c^ width of PS lamella.

## Supplementary Materials

The following are available online at https://www.mdpi.com/2079-4991/10/1/141/s1, Figure S1: Comparison of bright-field TEM at 200 kV and 60 kV acceleration voltage, Video S1: EFTEM-SI of PS-PMMA 50:50, Figure S2: EFTEM-SI image series of 70:30 PS:PMMA BCP., Figure S3: EFTEM-SI image series of 30:70 PS:PMMA BCP.
