# Supplementary material for: Characterisation of the PS-PMMA Interfaces in Microphase Separated Block Copolymer Thin Films by Analytical (S)TEM
Source: Nanomaterials (Basel). 2020 Jan 13;10(1):141. doi: 10.3390/nano10010141 (PMC7022429; doi:10.3390/nano10010141)
Supplement: Supplementary file 1 [file nanomaterials-10-00141-s001.zip › Brassat-Supplementary info.docx]

**Supplementary information**

**Characterisation of the PS-PMMA interfaces in microphase separated block copolymer thin films by analytical (S)TEM**

**Julius Bürger ^1,2,3^, Vinay S. Kunnathully ^1,2,3^, Daniel Kool ^1,2,3^, Jörg K. N. Lindner ^1,2,3^**

**and Katharina Brassat ^1,2,3,^***

^1^ Paderborn University, 33098 Paderborn, Germany

^2^ Institute of Lightweight Design with Hybrid Materials (ILH), Paderborn, Germany

^3^ Center for Optoelectronics and Photonics Paderborn (CeOPP), Paderborn, Germany

***** Correspondence: [katharina.brassat@uni-paderborn.de](mailto:katharina.brassat@uni-paderborn.de)

**1. Comparison of TEM bright-field imaging at acceleration voltages of 200 kV and 60 kV**

Figure S1 shows the difference of contrasts between PS and PMMA obtained in bright-field TEM analysis using acceleration voltages of 200 kV (left) and 60 kV (right), respectively. Both images show one hole in the *Quantifoil* TEM sample grid which is covered by a free standing membrane of the cylinder-forming BCP PS:PMMA 70:30. The insets show image sections at higher magnification. While barely any contrast between PMMA and PS domains is visible at 200 kV, the hexagonally arranged cylindrical PMMA domains are clearly visible at 60 kV. For both images almost similar illumination conditions were applied, i.e. 150 µm condenser lens aperture, broad beam and short integration times.


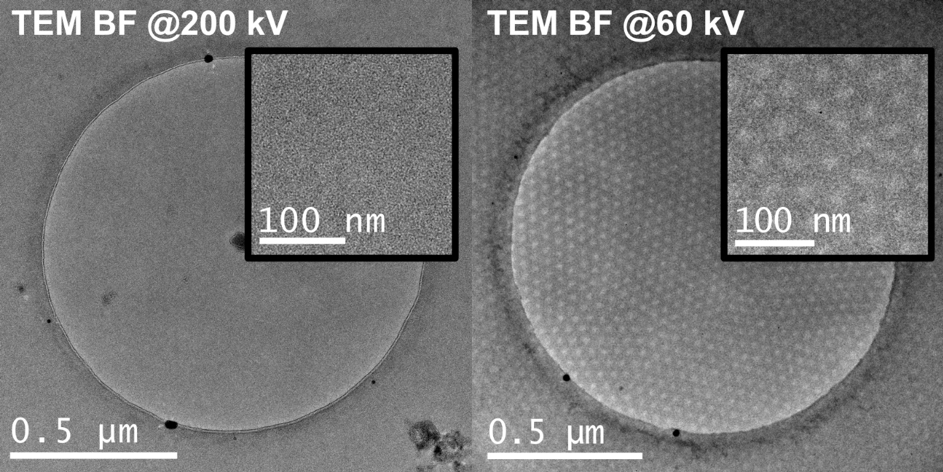
**Figure S1.** TEM bright-field imaging of PS-*b*-PMMA BCP thin films at 200 kV (left) and 60 kV (right). Insets show image sections at higher magnification.

**2. EFTEM-SI Video**

A video of the EFTEM-SI image series of the lamellae-forming 50:50 PS:PMMA BCP at different filtering states as shown in Figure 4 in the main body of the article can be downloaded (*EFTEM-SI_Lamella BCP_[-0,120] eV_10 eV slit.avi)* as a loop. The energy step for the sequent images is 10 eV. Thus, the whole video covers an energy range from -5 eV to 125 eV. The acquisition time per frame is 2 s. All images were manually realigned in a post-processing step.

**3. EFTEM-SI .BCP PS:PMMA 70:30 and 30:70**

In analogy to EFTEM-SI images of the lamellae-forming BCP PS:PMMA 50:50 in Figure 4 of the main body of the article, a filtered image series for the cylinder-forming BCPs of PS:PMMA 70:30 and 30:70 are acquired and displayed in Figure S2 and Figure S3, respectively.

EFTEM-SI images of the 70:30 BCP are captured with an acquisition time of 1 s and a filter slit width of 10 eV. In comparison, each frame of the EFTEM-SI series of the 30:70 BCP is obtained with an exposure time of 2 s and the same filter slit width. Even though the exposure time for the 70:30 BCP is 50 % lower, signal-to-noise ratio allows to analyse fine structures in the BCP thin film.


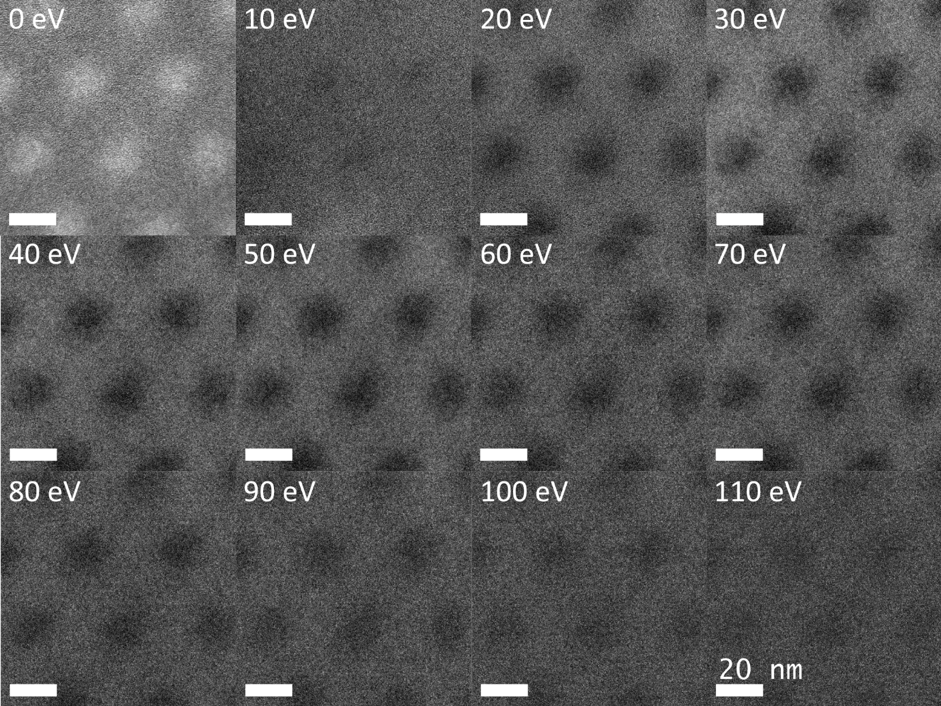
**Figure S2.** EFTEM-SI image series of 70:30 PS:PMMA BCP.


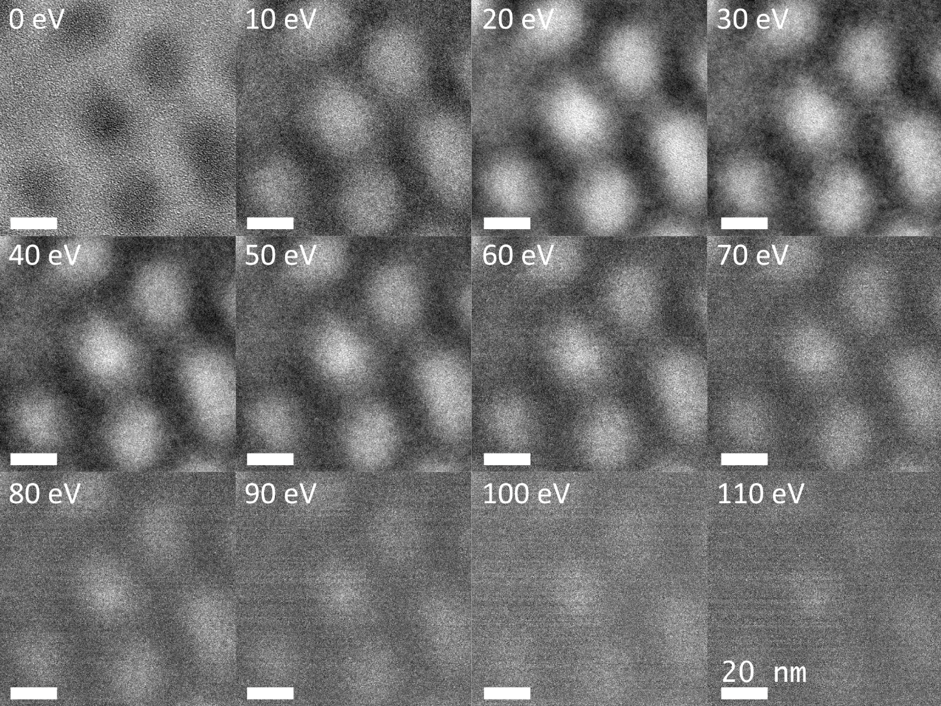
**Figure S3.** EFTEM-SI image series of 30:70 PS:PMMA BCP.

**4. EFTEM C-K elemental map: Experimental details and evaluation**

Figure 6 of the main manuscript shows an energy filtered TEM (EFTEM) carbon map using the characteristic carbon K edge in the lamellae-forming BCP PS:PMMA 50:50. The EFTEM map is created using the three-windows method, where a filter slit width of 20 eV is used to capture two pre-edge images centered at 252 eV and 272 eV energy offset and one post-edge image at 294 eV offset. Background fitting and subtraction is conducted using the two pre-edge images and assuming a power-law energy dependence. All underlying images are acquired at 60 kV with 60 s/frame.

For the evaluation let us call the PS rich lamella a PS lamella (I) and the PMMA rich lamella the PMMA lamella (II). The volume fraction of PS in lamella (I) is $\phi_{PS}^{I}=1-\phi$ and the volume fraction of PMMA amounts to $\phi_{PMMA}^{I}=\phi$, where ϕ is the volume fraction of non-phase separated polymer. Assuming that this volume fraction is identical to the volume fraction of PS in the PMMA lamella, one obtains for the volume fraction of PMMA and PS in the PMMA lamella (II) $\phi_{PMMA}^{II}=1-\phi$ and $\phi_{PS}^{II}=\phi$, Respectively. From the thickness map in Figure 5 (c) of the main manuscripts it follows that the thickness t_I_ of the PS lamellae (I) is 13.3% larger than the thickness t_II_ of the PMMA lamellae (II), neglecting the 2 % difference in mfps in PS and PMMA. Thus t_I_ = 1.133 t_II_. Since the ratio of carbon concentrations in PS and PMMA is 2:1, the expected carbon signal in lamella (I) is proportional to $C_{C}^{I}=t_{I}(2-\phi)$, while the carbon signal in lamella (II) is proportional to $C_{C}^{II}=t_{II}(1+2\phi)$, where the two proportionality factors are identical and depend on ionization cross sections and experimental conditions such as beam intensity, energy widths, collection angles and sensitivities. The experimentally determined ratio $C_{C}^{I}/C_{C}^{II}$ amounts to approximately 8/6=1.333. From this, the volume fraction ϕ of non-phase separated inclusions of PS in PMMA and PMMA in PS in the centre of lamellae is calculated to be 0.199.
